# The genome sequence of the variegated scallop, *Mimachlamys varia *(Linnaeus, 1758)

**DOI:** 10.12688/wellcomeopenres.19643.1

**Published:** 2023-07-14

**Authors:** Chris Fletcher, Mary E. Spencer Jones, Natural History Museum Genome Acquisition Lab, Darwin Tree of Life Barcoding collective, Wellcome Sanger Institute Tree of Life programme, Wellcome Sanger Institute Scientific Operations: DNA Pipelines collective, Tree of Life Core Informatics collective, Darwin Tree of Life Consortium

**Affiliations:** 1 Natural History Museum, London, England, UK

**Keywords:** Mimachlamys varia, variegated scallop, genome sequence, chromosomal, Pectinida

## Abstract

We present a genome assembly from an individual *Mimachlamys varia* (the variegated scallop; Mollusca; Bivalvia; Pectinida; Pectinidae). The genome sequence is 975.4 megabases in span. Most of the assembly is scaffolded into 19 chromosomal pseudomolecules. The mitochondrial genome has also been assembled and is 21.78 kilobases in length.

## Species taxonomy

Eukaryota; Metazoa; Eumetazoa; Bilateria; Protostomia; Spiralia; Lophotrochozoa; Mollusca; Bivalvia; Autobranchia; Pteriomorphia; Pectinida; Pectinoidea; Pectinidae; *Mimachlamys*; *Mimachlamys varia* (Linnaeus, 1758) (NCBI:txid50417).

## Background

**Figure 1.**
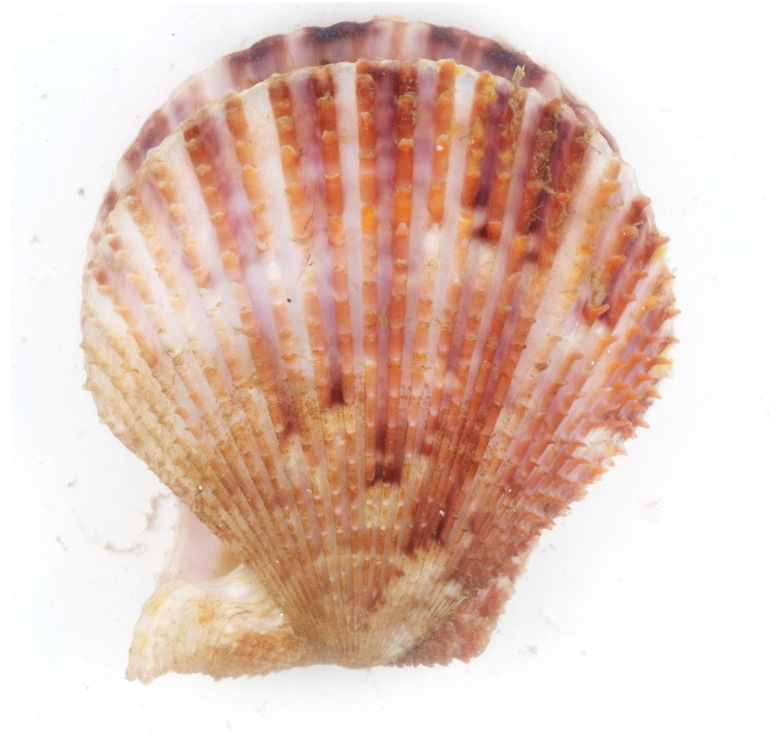
Photograph of the *Mimachlamys varia* (xbMimVari1) specimen used for genome sequencing.

The variegated scallop, *Mimachlamys varia,* is an inequivalve bivalve which is oval in shape with pronounced anterior ears and 25 to 35 prominent ribs, which have raised spatulate spines that increase in size towards the margin ( Hayward & Ryland, 2017). Adults can usually reach up to 60 mm and are very variable in colour, ranging from off-white to yellow, orange, brown and purple or a combination of these colours as demonstrated by the sequenced specimen ( Figure 1).

Under laboratory conditions, larvae prefer to settle in sheltered areas of low light and vertical or sloping surfaces, with additional substrate increasing settlement ( De la Roche *et al.*, 2009; Rodhouse & Burnell, 1979). Once metamorphosis has occurred, juveniles mature as males but may change their sex several times as adults ( Hayward & Ryland, 2017). *M. varia* attaches to substrate using byssus threads ( Duncan *et al.*, 2016) and can be found in high numbers in association with *Modiolus modiolus* beds alongside ascidians, hydroids and other epifaunal species ( JNCC, 2022). Indeed, the specimen collected for genome sequencing was found attached to the underside of a small oblong marina buoy, in amongst similar epifauna.

*M. varia* is common in inner shore habitats around Britain and Ireland, northern Europe, the Mediterranean and West Africa. In Europe, the main commercial species of scallop are *Pecten maximus* and *Aquipecten opecularis*; however, *M. varia* is also landed alongside *A. opecularis* in France and Spain ( Duncan *et al.*, 2016). *M. varia* is also regarded as a useful tool for biomonitoring of water quality in ports and harbours on the coast of France ( Barbarin *et al.*, 2022; Breitwieser *et al.*, 2018; Breitwieser *et al.*, 2020).

Various genetic studies of *M. varia* have been published ( Arias *et al.*, 2009b; Arias *et al.*, 2009a; Beajmont & Beveridge, 1984; Breitwieser *et al.*, 2018; Fernandez-Moreno *et al.*, 2008), most notably the sequencing of its transcriptome ( Viricel *et al.*, 2018) and whole mitochondrial genome ( Malkócs *et al.*, 2022). Here we present the chromosomally complete genome sequence for *M. varia*, sequenced as part of the Darwin Tree of Life Project. It is hoped that this will provide further insight into the biology, ecology and evolution of *M. varia* and other pectinid bivalves.

## Genome sequence report

The genome was sequenced from one *Mimachlamys varia* specimen ( Figure 1) collected from Haslar Marina, Gosport, UK (50.79, –1.12). A total of 44-fold coverage in Pacific Biosciences single-molecule HiFi long reads was generated. Primary assembly contigs were scaffolded with chromosome conformation Hi-C data. Manual assembly curation corrected 29 missing joins or mis-joins and removed 8 haplotypic duplications, reducing the assembly length by 0.56% and the scaffold number by 8.79%.

**Figure 2.**
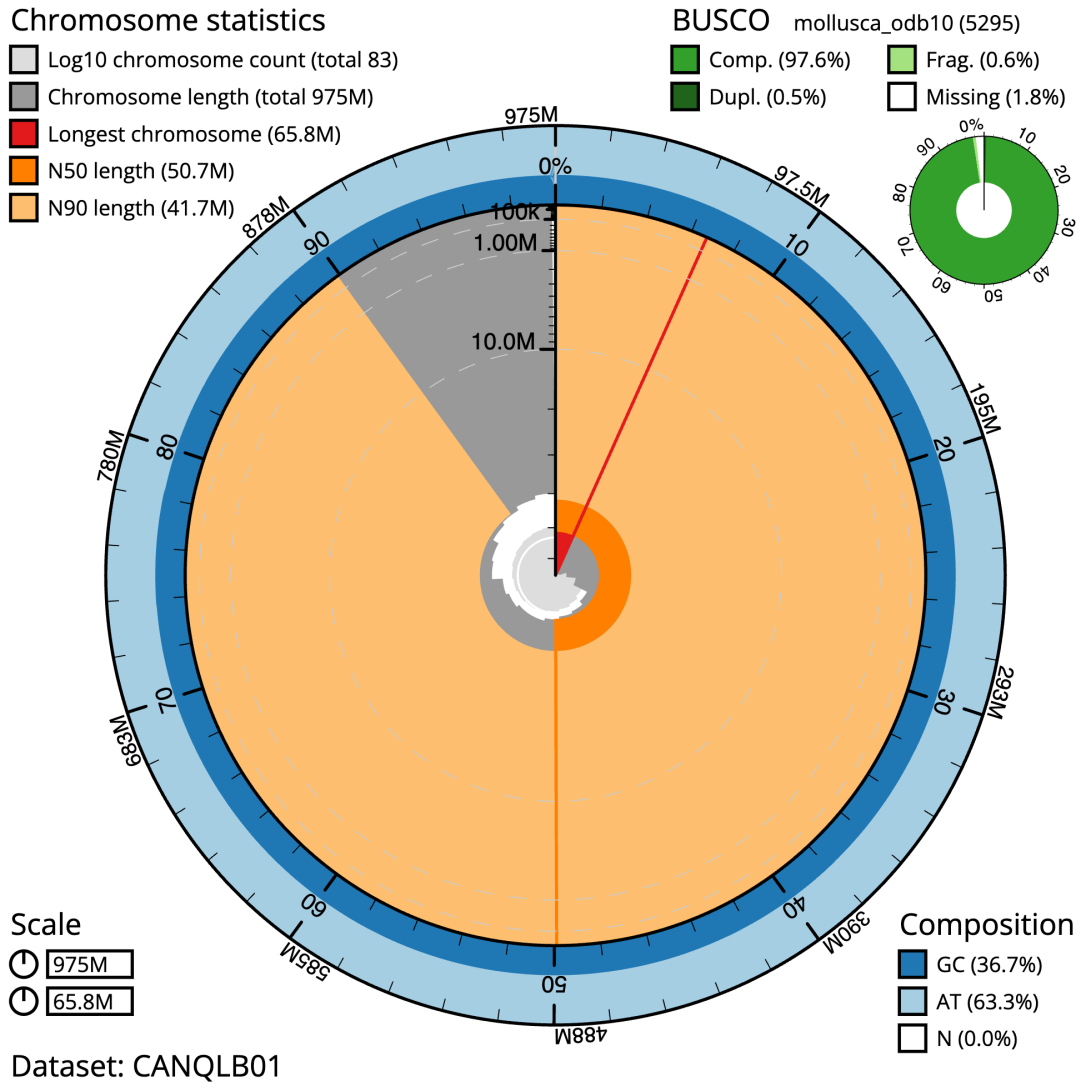
Genome assembly of *Mimachlamys varia*, xbMimVari1.1: metrics. The BlobToolKit Snailplot shows N50 metrics and BUSCO gene completeness. The main plot is divided into 1,000 size-ordered bins around the circumference with each bin representing 0.1% of the 975,381,475 bp assembly. The distribution of scaffold lengths is shown in dark grey with the plot radius scaled to the longest scaffold present in the assembly (65,822,114 bp, shown in red). Orange and pale-orange arcs show the N50 and N90 scaffold lengths (50,749,371 and 41,658,273 bp), respectively. The pale grey spiral shows the cumulative scaffold count on a log scale with white scale lines showing successive orders of magnitude. The blue and pale-blue area around the outside of the plot shows the distribution of GC, AT and N percentages in the same bins as the inner plot. A summary of complete, fragmented, duplicated and missing BUSCO genes in the mollusca_odb10 set is shown in the top right. An interactive version of this figure is available at https://blobtoolkit.genomehubs.org/view/xbMimVari1.1/dataset/CANQLB01/snail.

The final assembly has a total length of 975.4 Mb in 82 sequence scaffolds with a scaffold N50 of 50.7 Mb ( Table 1). Most (99.76%) of the assembly sequence was assigned to 19 chromosomal-level scaffolds. Chromosome-scale scaffolds confirmed by the Hi-C data are named in order of size ( Figure 2– Figure 5; Table 2). While not fully phased, the assembly deposited is of one haplotype. Contigs corresponding to the second haplotype have also been deposited. The mitochondrial genome was also assembled and can be found as a contig within the multifasta file of the genome submission.

**Table 1.** Genome data for *Mimachlamys varia*, xbMimVari1.1.

Project accession data		
Assembly identifier	xbMimVari1.1	
Species	*Mimachlamys varia*	
Specimen	xbMimVari1	
NCBI taxonomy ID	50417	
BioProject	PRJEB57314	
BioSample ID	SAMEA8534354	
Isolate information	xbMimVari1, muscle tissue (DNA sequencing, Hi-C scaffolding and RNA sequencing)	
Assembly metrics *		*Benchmark*
Consensus quality (QV)	64.4	*≥ 50*
*k*-mer completeness	100%	*≥ 95%*
BUSCO **	C:97.6%[S:97.1%,D:0.5%],F:0.6%, M:1.8%,n:5,295	*C ≥ 95%*
Percentage of assembly mapped to chromosomes	99.76%	*≥ 95%*
Sex chromosomes	-	*localised homologous pairs*
Organelles	Mitochondrial genome assembled	*complete single alleles*
Raw data accessions		
PacificBiosciences SEQUEL II	ERR10480598, ERR10480599, ERR10480600	
Hi-C Illumina	ERR10466821	
PolyA RNA-Seq Illumina	ERR10908612	
Genome assembly		
Assembly accession	GCA_947623455.1	
*Accession of alternate haplotype*	GCA_947622925.1	
Span (Mb)	975.4	
Number of contigs	171	
Contig N50 length (Mb)	19.4	
Number of scaffolds	82	
Scaffold N50 length (Mb)	50.7	
Longest scaffold (Mb)	65.9	

* Assembly metric benchmarks are adapted from column VGP-2020 of “Table 1: Proposed standards and metrics for defining genome assembly quality” from ( Rhie *et al.*, 2021). ** BUSCO scores based on the mollusca_odb10 BUSCO set using v5.3.2. C = complete [S = single copy, D = duplicated], F = fragmented, M = missing, n = number of orthologues in comparison. A full set of BUSCO scores is available at https://blobtoolkit.genomehubs.org/view/xbMimVari1.1/dataset/CANQLB01/busco.

**Figure 3.**
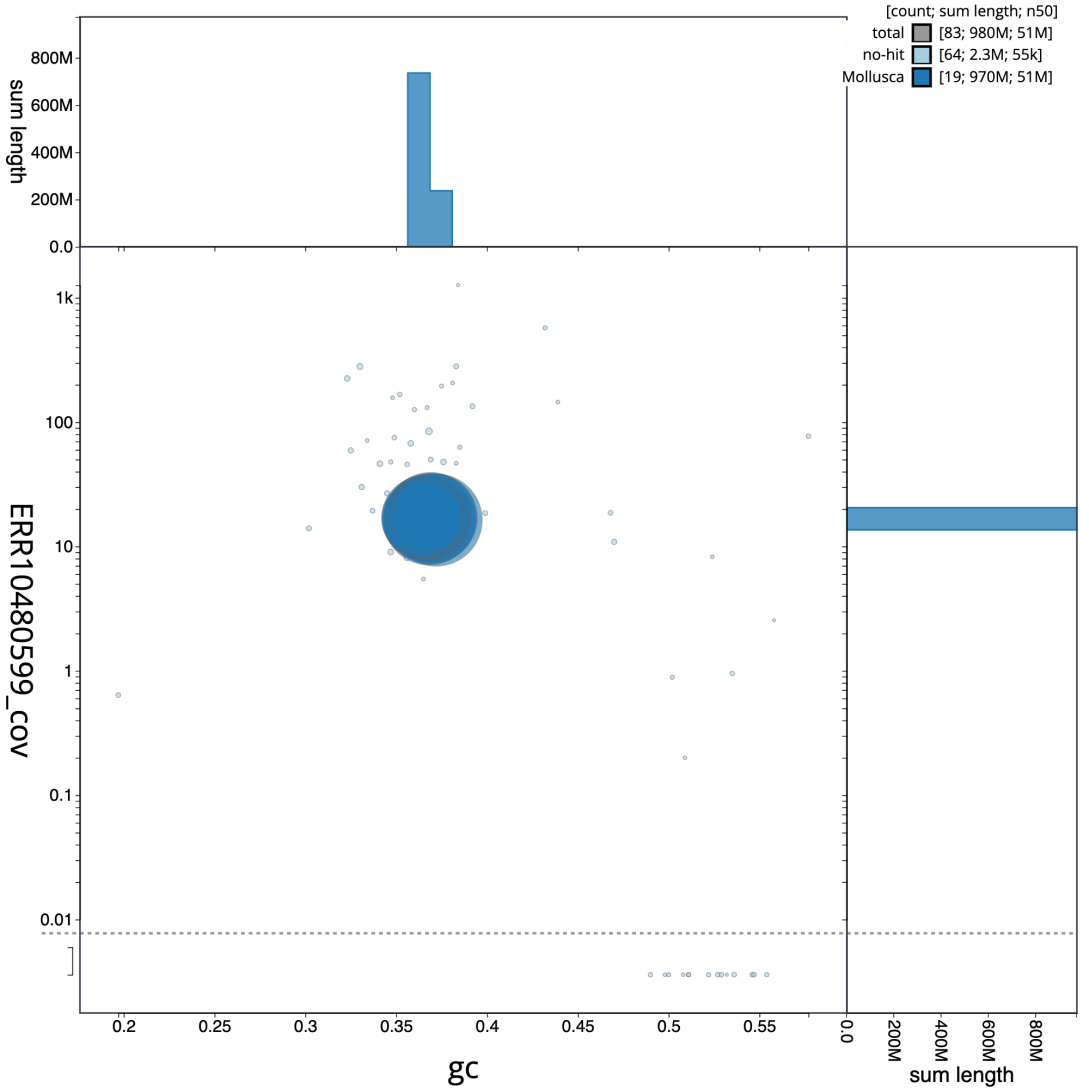
Genome assembly of *Mimachlamys varia*, xbMimVari1.1: BlobToolKit GC-coverage plot. Scaffolds are coloured by phylum. Circles are sized in proportion to scaffold length. Histograms show the distribution of scaffold length sum along each axis. An interactive version of this figure is available at https://blobtoolkit.genomehubs.org/view/xbMimVari1.1/dataset/CANQLB01/blob.

**Figure 4.**
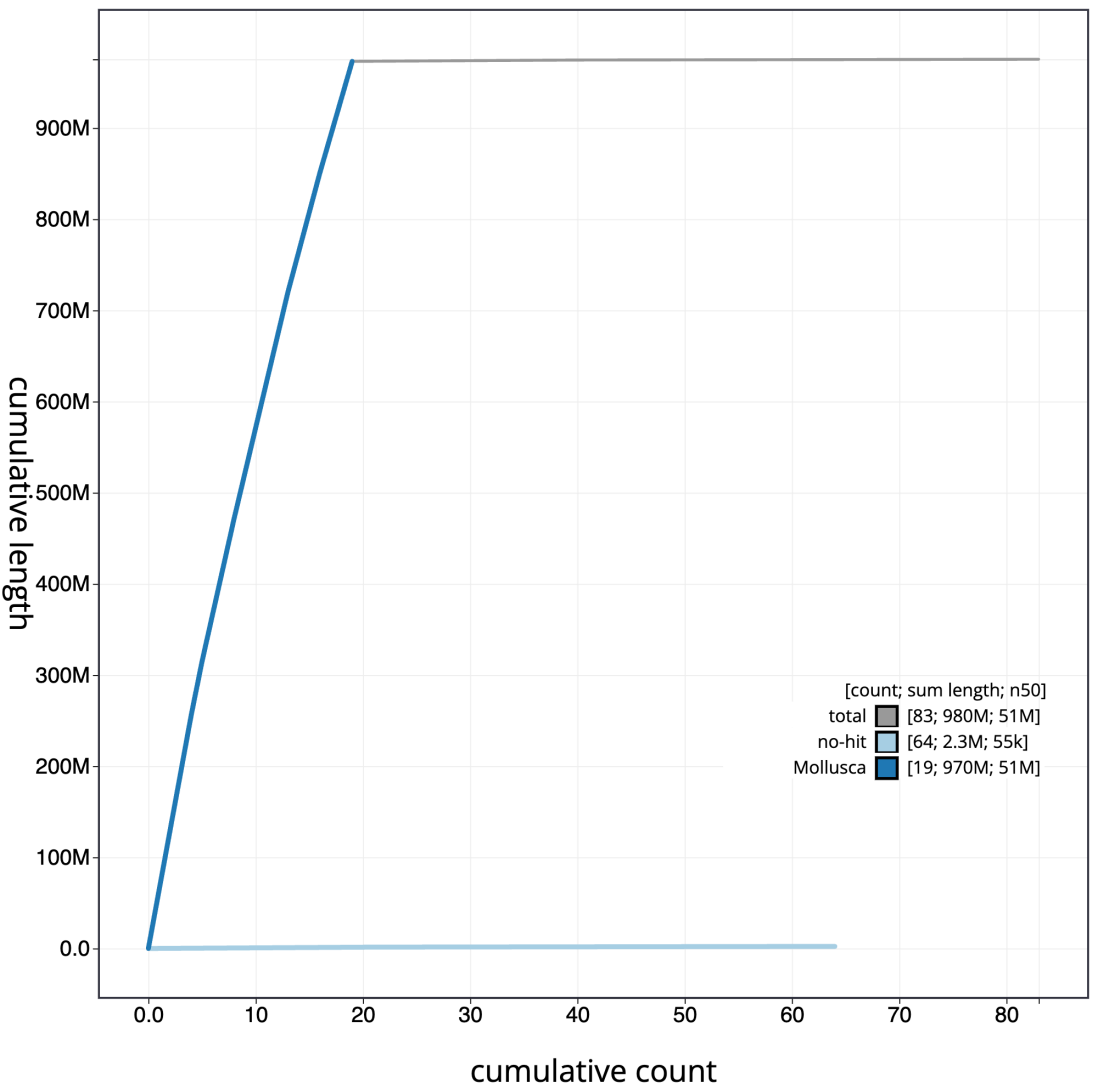
Genome assembly of *Mimachlamys varia*, xbMimVari1.1: BlobToolKit cumulative sequence plot. The grey line shows cumulative length for all scaffolds. Coloured lines show cumulative lengths of scaffolds assigned to each phylum using the buscogenes taxrule. An interactive version of this figure is available at https://blobtoolkit.genomehubs.org/view/xbMimVari1.1/dataset/CANQLB01/cumulative.

**Figure 5.**
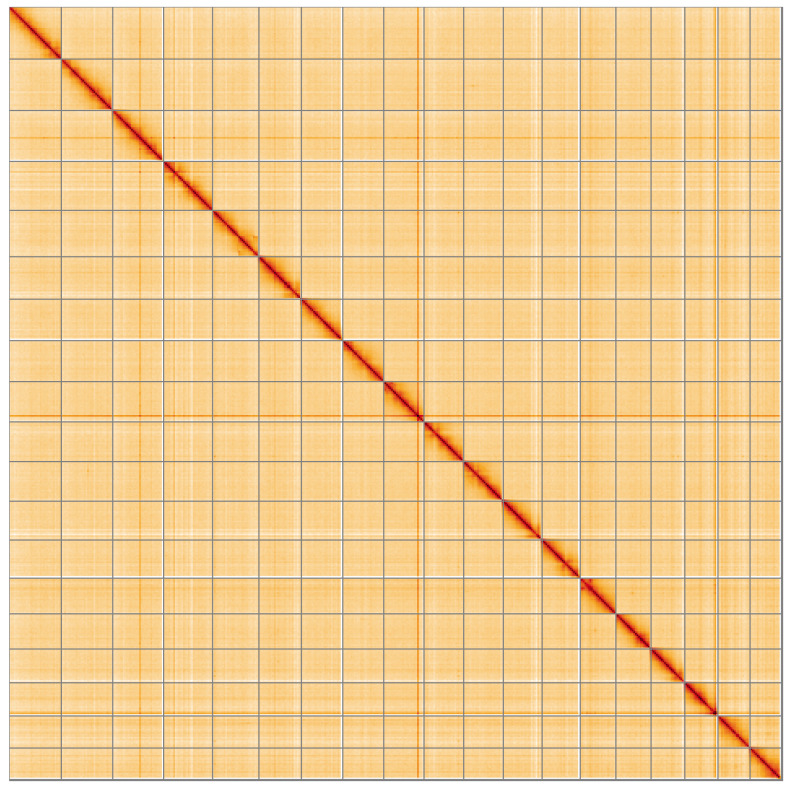
Genome assembly of *Mimachlamys varia*, xbMimVari1.1: Hi-C contact map of the xbMimVari1.1 assembly, visualised using HiGlass. Chromosomes are shown in order of size from left to right and top to bottom. An interactive version of this figure may be viewed at https://genome-note-higlass.tol.sanger.ac.uk/l/?d=W3n8QQetR4WPxi2j_7zzcg.

**Table 2.** Chromosomal pseudomolecules in the genome assembly of *Mimachlamys varia*, xbMimVari1.

INSDC accession	Chromosome	Length (Mb)	GC%
OX392527.1	1	65.82	37.0
OX392528.1	2	64.71	37.0
OX392529.1	3	64.13	37.0
OX392530.1	4	61.64	37.0
OX392531.1	5	58.42	36.5
OX392532.1	6	53.44	36.5
OX392533.1	7	52.18	36.5
OX392534.1	8	51.61	36.5
OX392535.1	9	50.75	36.5
OX392536.1	10	49.95	36.5
OX392537.1	11	49.9	36.5
OX392538.1	12	48.95	36.5
OX392539.1	13	48.05	36.5
OX392540.1	14	44.87	37.0
OX392541.1	15	44.36	36.5
OX392542.1	16	42.53	36.5
OX392543.1	17	41.66	36.5
OX392544.1	18	40.52	36.5
OX392545.1	19	39.6	36.5
OX392546.1	MT	0.02	43.0

The estimated Quality Value (QV) of the final assembly is 64.4 with *k*-mer completeness of 100%, and the assembly has a BUSCO v5.3.2 completeness of 97.6% (single = 97.1%, duplicated = 0.5%), using the mollusca_odb10 reference set ( *n* = 5,295).

Metadata for specimens, spectral estimates, sequencing runs, contaminants and pre-curation assembly statistics can be found at https://links.tol.sanger.ac.uk/species/50417.

## Methods

### Sample acquisition and nucleic acid extraction

The *Mimachlamys varia* specimen selected for genome sequencing was individual xbMimVari1, collected from the Haslar Marina, Gosport, UK (latitude 50.79, longitude –1.12) on 2020-09-24. The specimen was picked by hand from the underside of a small buoy attached to the marina pontoon by Chris Fletcher and Mary E. Spencer Jones (both Natural History Museum). The specimen was identified by Chris Fletcher and tissue extracted from the adductor muscle was preserved in liquid nitrogen. The remainder of the specimen was preserved in 80% ethanol and stored at the Natural History Museum, London.

DNA was extracted at the Tree of Life laboratory, Wellcome Sanger Institute (WSI). The xbMimVari1 sample was weighed and dissected on dry ice with tissue set aside for Hi-C sequencing. Muscle tissue was cryogenically disrupted to a fine powder using a Covaris cryoPREP Automated Dry Pulveriser, receiving multiple impacts. High molecular weight (HMW) DNA was extracted using the Qiagen MagAttract HMW DNA extraction kit. HMW DNA was sheared into an average fragment size of 12–20 kb in a Megaruptor 3 system with speed setting 30. Sheared DNA was purified by solid-phase reversible immobilisation using AMPure PB beads with a 1.8X ratio of beads to sample to remove the shorter fragments and concentrate the DNA sample. The concentration of the sheared and purified DNA was assessed using a Nanodrop spectrophotometer and Qubit Fluorometer and Qubit dsDNA High Sensitivity Assay kit. Fragment size distribution was evaluated by running the sample on the FemtoPulse system.

RNA was extracted from muscle tissue of xbMimVari1 in the Tree of Life Laboratory at the WSI using TRIzol, according to the manufacturer’s instructions. RNA was then eluted in 50 μl RNAse-free water and its concentration assessed using a Nanodrop spectrophotometer and Qubit Fluorometer using the Qubit RNA Broad-Range (BR) Assay kit. Analysis of the integrity of the RNA was done using Agilent RNA 6000 Pico Kit and Eukaryotic Total RNA assay.

### Sequencing

Pacific Biosciences HiFi circular consensus DNA sequencing libraries were constructed according to the manufacturers’ instructions *.* Poly(A) RNA-Seq libraries were constructed using the NEB Ultra II RNA Library Prep kit. DNA and RNA sequencing was performed by the Scientific Operations core at the WSI on Pacific Biosciences SEQUEL II (HiFi) and Illumina NovaSeq 6000 (RNA-Seq) instruments. Hi-C data were also generated from $HIC_TISSUE tissue of xbMimVari1 using the Arima2 kit and sequenced on the Illumina NovaSeq 6000 instrument.

### Genome assembly, curation and evaluation

Assembly was carried out with Hifiasm ( Cheng *et al.*, 2021) and haplotypic duplication was identified and removed with purge_dups ( Guan *et al.*, 2020). The assembly was then scaffolded with Hi-C data ( Rao *et al.*, 2014) using YaHS. The assembly was checked for contamination and corrected using the gEVAL system ( Chow *et al.*, 2016) as described previously ( Howe *et al.*, 2021). Manual curation was performed using gEVAL, HiGlass ( Kerpedjiev *et al.*, 2018) and Pretext ( Harry, 2022). The mitochondrial genome was assembled using MitoHiFi ( Uliano-Silva *et al.*, 2022), which runs MitoFinder ( Allio *et al.*, 2020) or MITOS ( Bernt *et al.*, 2013) and uses these annotations to select the final mitochondrial contig and to ensure the general quality of the sequence.

A Hi-C map for the final assembly was produced using bwa-mem2 ( Vasimuddin *et al.*, 2019) in the Cooler file format ( Abdennur & Mirny, 2020). To assess the assembly metrics, the *k*-mer completeness and QV consensus quality values were calculated in Merqury ( Rhie *et al.*, 2020). This work was done using Nextflow ( Di Tommaso *et al.*, 2017) DSL2 pipelines “sanger-tol/readmapping” ( Surana *et al.*, 2023a) and “sanger-tol/genomenote” ( Surana *et al.*, 2023b). The genome was analysed within the BlobToolKit environment ( Challis *et al.*, 2020) and BUSCO scores ( Manni *et al.*, 2021; Simão *et al.*, 2015) were calculated.

Table 3 contains a list of relevant software tool versions and sources.

**Table 3.** Software tools: versions and sources.

Software tool	Version	Source
BlobToolKit	4.1.5	https://github.com/blobtoolkit/ blobtoolkit
BUSCO	5.3.2	https://gitlab.com/ezlab/busco
gEVAL	N/A	https://geval.org.uk/
Hifiasm	0.16.1-r375	https://github.com/chhylp123/ hifiasm
HiGlass	1.11.6	https://github.com/higlass/higlass
Merqury	MerquryFK	https://github.com/thegenemyers/ MERQURY.FK
MitoHiFi	2	https://github.com/marcelauliano/ MitoHiFi
PretextView	0.2	https://github.com/wtsi-hpag/ PretextView
purge_dups	1.2.3	https://github.com/dfguan/purge_ dups
sanger-tol/ genomenote	v1.0	https://github.com/sanger-tol/ genomenote
sanger-tol/ readmapping	1.1.0	https://github.com/sanger-tol/ readmapping/tree/1.1.0
YaHS	1.1a.2	https://github.com/c-zhou/yahs

### Wellcome Sanger Institute – Legal and Governance

The materials that have contributed to this genome note have been supplied by a Darwin Tree of Life Partner. The submission of materials by a Darwin Tree of Life Partner is subject to the **‘Darwin Tree of Life Project Sampling Code of Practice’**, which can be found in full on the Darwin Tree of Life website here. By agreeing with and signing up to the Sampling Code of Practice, the Darwin Tree of Life Partner agrees they will meet the legal and ethical requirements and standards set out within this document in respect of all samples acquired for, and supplied to, the Darwin Tree of Life Project.

Further, the Wellcome Sanger Institute employs a process whereby due diligence is carried out proportionate to the nature of the materials themselves, and the circumstances under which they have been/are to be collected and provided for use. The purpose of this is to address and mitigate any potential legal and/or ethical implications of receipt and use of the materials as part of the research project, and to ensure that in doing so we align with best practice wherever possible. The overarching areas of consideration are:

•   Ethical review of provenance and sourcing of the material

•   Legality of collection, transfer and use (national and international)

Each transfer of samples is further undertaken according to a Research Collaboration Agreement or Material Transfer Agreement entered into by the Darwin Tree of Life Partner, Genome Research Limited (operating as the Wellcome Sanger Institute), and in some circumstances other Darwin Tree of Life collaborators.

## Data Availability

European Nucleotide Archive: *Mimachlamys varia*. Accession number PRJEB57314; https://identifiers.org/ena.embl/PRJEB57314. ( Wellcome Sanger Institute, 2023) The genome sequence is released openly for reuse. The *Mimachlamys varia* genome sequencing initiative is part of the Darwin Tree of Life (DToL) project. All raw sequence data and the assembly have been deposited in INSDC databases. The genome will be annotated using available RNA-Seq data and presented through the Ensembl pipeline at the European Bioinformatics Institute. Raw data and assembly accession identifiers are reported in Table 1.

## Author information

Members of the Natural History Museum Genome Acquisition Lab are listed here:

https://doi.org/10.5281/zenodo.4790042.

Members of the Darwin Tree of Life Barcoding collective are listed here: https://doi.org/10.5281/zenodo.4893703.

Members of the Wellcome Sanger Institute Tree of Life programme are listed here: https://doi.org/10.5281/zenodo.4783585.

Members of Wellcome Sanger Institute Scientific Operations: DNA Pipelines collective are listed here: https://doi.org/10.5281/zenodo.4790455.

Members of the Tree of Life Core Informatics collective are listed here: https://doi.org/10.5281/zenodo.5013541.

Members of the Darwin Tree of Life Consortium are listed here: https://doi.org/10.5281/zenodo.4783558.
