# Supplementary material for: The genome sequence of the variegated scallop, Mimachlamys varia (Linnaeus, 1758)
Source: Wellcome Open Res. 2023 Jul 14;8:307. [Version 1] doi: 10.12688/wellcomeopenres.19643.1 (PMC11252643; doi:10.12688/wellcomeopenres.19643.1)
Supplement: Peer review history

# Reviewer report: Reviewer response for version 1

**DOI:** 10.21956/wellcomeopenres.21760.r85609

**Published:** 2024-07-16

**Authors:** Stéphane Coupé

**Affiliations:** 1 Universite de Toulon, Toulon, Provence-Alpes-Côte d'Azur, France

Fletcher and Jones produced an additional bivalve genome sequence, which is of particular importance for comparative genomic analysis and conservation biology, especially given the current and growing threats on biodiversity.

 The background section gives enough relevant information about the species for a data note.

 The genome sequence report is clear and straightforward. It obviously states the quality of the obtained genome sequence.

 The methods section evidences that the genome sequence has been obtained with gold-standard techniques and bioinformatic analyses. It results in a well assembled genome, that remains to be annotated.

 The figures and tables are clear and informative.

**As a consequence I think this data note is worth indexing.**

 I have minor comments that the authors may consider :

Results:

 What were the fragment size and mean quality distributions?

 Did you assess the repetitive DNA?

Methods:

 How many mg of tissue did you use?

 Tissue protocol for hi-C sequencing should be detailed, unless not required for a data note.

 What kind of manual corrections have you performed, and how did you proceed?

 How did you filter raw sequences? For instance you referred to contamination, could you clarify?

 Did you use some reference genome for assembly or verification ? If yes, please be precise and justify.

 Can you clarify the reference dataset used for BUSCO analysis?

 Why mention RNA sequencing if it was not used for genome assembly? If yes, please be precise.

Are sufficient details of methods and materials provided to allow replication by others?

Yes

Is the rationale for creating the dataset(s) clearly described?

Yes

Are the datasets clearly presented in a useable and accessible format?

Yes

Are the protocols appropriate and is the work technically sound?

Yes

Reviewer Expertise:

Population genomics

I confirm that I have read this submission and believe that I have an appropriate level of expertise to confirm that it is of an acceptable scientific standard.

# Reviewer report: Reviewer response for version 1

**DOI:** 10.21956/wellcomeopenres.21760.r85612

**Published:** 2024-06-19

**Authors:** Kyle E McElroy

**Affiliations:** 1 Department of Ecology, Evolutionary, and Organismal Biology, Iowa State University, Ames, Iowa, USA

The authors present, as part of the Darwin Tree of Life Project, a reference genome assembly for the scallop *Mimachlamys varia*. The genome was assembled with PacBio HiFi reads and scaffolded with Hi-C sequencing. It will be annotated in future work, but the authors also produced RNA-seq from the same reference animal for those efforts. The genome is assembled into 19 chromosomes and appears to be of exceedingly high quality and completeness by several metrics. The complete mitochondria genome is also reported. I think this assembly will be valuable to many researchers interested in comparative bivalve/mollusk genomics. I’m glad to see more scallop genomes assemblies produced by this research group. My main critiques (below) are largely concerning additional methodological details that should be included in this report. These critiques are quite small and should be easy for the authors to address.

 Major criticism:

 The degree of detail for the HWM DNA and RNA extractions is much greater than generating the input for Hi-C sequencing. While Hi-C sequencing is mentioned in the “Sequencing” section of the Methods, I think some more detail about the tissue and library prep process is warranted in “Sample acquisition and nucleic acid extraction” section. For example, what endonuclease was used to fragment crosslinked DNA and how was the library quality assessed? Also, in this section I noticed “$HIC_TISSUE” – I think this might be remnant text from a manuscript template?

 For the Hi-C and RNA-seq, it should be noted that the libraries are 2x150 bp reads. Also, the total amount of sequence data generated from these runs should be reported. The HiFi output was reported as “44-fold coverage” but I also think something more along the lines of total bases and number of reads generated should be included.

 Minor comments/questions:

 “Inequivalve bivalve” – I think this term should be defined.

 “The specimen was identified by Chris Fletcher” – Was there any morphological criteria or molecular analysis done to confirm the species ahead of sequencing?

 Is the Ultra II RNA Library Prep Kit a stranded library prep? Also, if it is not beyond the scope of detailing the genome, I think some detail about the quality of this data would be useful. Mainly, how much of it maps back to the genome assembly?

 It might be worth noting that 19 chromosomes are expected based on other chromosome-level assemblies of scallops and karyotypic analyses. Unless such comparison is beyond the scope of a Data Note in Wellcome Open Research.

 Did the MitoHiFi pipeline return a circular molecule, is that reported in the output?

 I know the rest of the annotation is yet to be done, but the mitochondrial genome assembly pipeline includes an automated annotation. Will this be the “official” annotation, if so, should those results be included here?

Are sufficient details of methods and materials provided to allow replication by others?

Yes

Is the rationale for creating the dataset(s) clearly described?

Yes

Are the datasets clearly presented in a useable and accessible format?

Yes

Are the protocols appropriate and is the work technically sound?

Yes

Reviewer Expertise:

Evolutionary biology, genomics, genome evolution, bivalves, gene-family evolution, opsins, asexual reproduction, transposable elements

I confirm that I have read this submission and believe that I have an appropriate level of expertise to confirm that it is of an acceptable scientific standard, however I have significant reservations, as outlined above.

# Reviewer report: Reviewer response for version 1

**DOI:** 10.21956/wellcomeopenres.21760.r63027

**Published:** 2023-07-24

**Authors:** José María Martín-Durán

**Affiliations:** 1 School of Biological and Behavioural Sciences, Queen Mary University of London, London, UK

This manuscript reports the chromosome-scale genome assembly of the bivalve *Mimachlamys varia*, a common member of the fauna on the shores of the UK, North Atlantic and Mediterranean seas. Produced with gold-standard methods of the DToL, this is a high-quality assembly that will be a valuable resource for many research fields, from evolutionary biology to ecology and marine biology.

Are sufficient details of methods and materials provided to allow replication by others?

Yes

Is the rationale for creating the dataset(s) clearly described?

Yes

Are the datasets clearly presented in a useable and accessible format?

Yes

Are the protocols appropriate and is the work technically sound?

Yes

Reviewer Expertise:

Animal genomics, marine invertebrate evo-devo

I confirm that I have read this submission and believe that I have an appropriate level of expertise to confirm that it is of an acceptable scientific standard.
